# A Degenerate Peptide Library Approach to Reveal Sequence Determinants of Methyllysine-Driven Protein Interactions

**DOI:** 10.3389/fcell.2020.00241

**Published:** 2020-04-09

**Authors:** Ariana Kupai, Robert M. Vaughan, Bradley M. Dickson, Scott B. Rothbart

**Affiliations:** Center for Epigenetics, Van Andel Institute, Grand Rapids, MI, United States

**Keywords:** lysine methylation, reader domains, functional proteomics, non-histone proteins, lysine-orientated peptide libraries

## Abstract

Lysine methylation facilitates protein-protein interactions through the activity of methyllysine (Kme) “reader” proteins. Functions of Kme readers have historically been studied in the context of histone interactions, where readers aid in chromatin-templated processes such as transcription, DNA replication and repair. However, there is growing evidence that Kme readers also function through interactions with non-histone proteins. To facilitate expanded study of Kme reader activities, we developed a high-throughput binding assay to reveal the sequence determinants of Kme-driven protein interactions. The assay queries a degenerate methylated lysine-oriented peptide library (Kme-OPL) to identify the key residues that modulate reader binding. The assay recapitulated methyl order and amino acid sequence preferences associated with histone Kme readers. The assay also revealed methylated sequences that bound Kme readers with higher affinity than histones. Proteome-wide scoring was applied to assay results to help prioritize future study of Kme reader interactions. The platform was also used to design sequences that directed specificity among closely related reader domains, an application which may have utility in the development of peptidomimetic inhibitors. Furthermore, we used the platform to identify binding determinants of site-specific histone Kme antibodies and surprisingly revealed that only a few amino acids drove epitope recognition. Collectively, these studies introduce and validate a rapid, unbiased, and high-throughput binding assay for Kme readers, and we envision its use as a resource for expanding the study of Kme-driven protein interactions.

## Introduction

Lysines can be mono-, di-, or tri-methylated on the sidechain e-amino group (Ambler and Rees, 1959; Alix et al., 1979) and this post-translational modification can be “read” by proteins that contain methyllysine (Kme) binding domains (e.g., chromo, Tudor, MBT, PHD, etc.) (Liu et al., 2012). The first discovered Kme reader was heterochromatin protein 1 (HP1), whose chromodomain binds tri-methylated lysine 9 on histone H3 (H3K9me3) and facilitates HP1-mediated gene silencing (Bannister et al., 2001). Since this turn of the century discovery, more than 200 Kme reader proteins have been identified (Liu et al., 2012). Nearly all of these proteins have been studied as histone Kme readers and have been linked to various chromatin-associated functions like transcriptional regulation (Wozniak and Strahl, 2014), DNA repair (Botuyan et al., 2006) and DNA replication (Kuo et al., 2012).

The study of Kme reader-protein interactions is expanding beyond histones (Cornett et al., 2019). For example, M-phase phosphoprotein 8 (MPP8) is a Kme reader that, like HP1, was linked to gene silencing through recognition of H3K9me3 through its chromodomain (Kokura et al., 2010). MPP8 also functions in gene regulation through interactions with non-histone proteins like DNA methyltransferase 3a (DNMT3a) (Chang et al., 2011) and activating transcription factor 7-interacting protein 1 (ATF7IP) (Tsusaka et al., 2018). Other Kme readers, including HP1 (Liu et al., 2013), also have reported non-histone interactions (Cui et al., 2012; Ferry et al., 2017). Lysine methylation has been detected on over 3,000 unique human proteins (Hornbeck et al., 2015) but functions associated with Kmes are limited. This gap in knowledge has persisted in part because few technologies can directly associate proteins with Kme readers (Ong and Mann, 2006; Guo et al., 2014).

**FIGURE 1:**
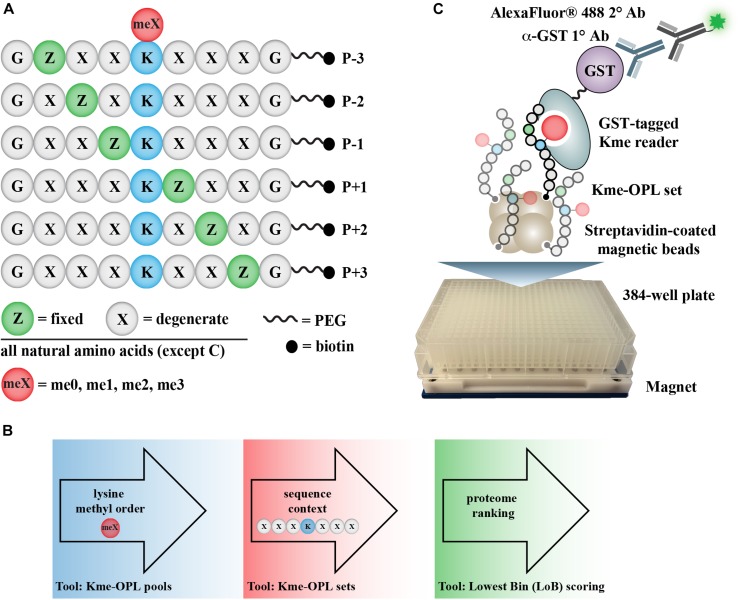
Kme-OPL assay overview. **(A)** Design of Kme-OPL. **(B)** Schematic of assay workflow. **(C)** Cartoon of the magnetic bead pulldown assay developed for screening the activities of Kme readers. PEG, polyethylene glycol.

Here, we describe the development of a high-throughput assay for rapid, *in vitro* determination of where a Kme reader may bind in the proteome. The method identifies Kme-driven interactions by screening a Kme reader against a methyllysine-oriented peptide library (Kme-OPL) (Figure 1A). The OPL synthetic strategy is modified from the development of positional scanning peptide libraries (Houghten et al., 1991), and variations have been successfully applied to the study of other signaling processes, including phosphorylation and arginine methylation (Creixell et al., 2015; Gayatri et al., 2016). The degeneracy of the peptide library allows for the survey of all amino acid sequence combinations (excluding cysteine) minus to plus three (P-3/+3) from a central Kme. The assay informs on methyl order (Kme0, Kme1, Kme2, Kme3) preference and amino acid context, two key determinants of Kme reader interactions. Amino acid preferences are used to rank all lysine-centered motifs in the human proteome for each Kme reader, and these data are made available as a communal resource to help facilitate the identification of new Kme driven-protein interactions (Figure 1B). Additionally, Kme-specific antibodies can be used in place of Kme readers in this assay. Here, we report the use of the Kme-OPL assay for detecting the preferred methyl order of binding for multiple Kme readers, determining the optimal amino acid context for Kme reader binding, and revealing the binding determinants of histone Kme-specific antibodies.

## Materials and Methods

### Recombinant Protein Production

Plasmids encoding N-terminal GST fusions of each reader domain (Supplementary Table S1) were transformed into BL21 *E. coli* and protein expression was induced with 0.5 mM IPTG at 16°C for 6–16 h. Induced bacterial pellets were suspended in 30 mL cold 1× PBS supplemented with 1 mM DTT and 1 mM PMSF. Next, bacteria were incubated with lysozyme (Thermo #89833 LOT#ta262343) and 1 μL of Pierce universal nuclease (Pierce #88702 LOT#00775219) on ice for 30 min followed by 3 rounds of sonication (30 s sonication with 10 s rest, all on ice) using a Qsonica ultrasonic processor (500 W 20 kHz with 1/8” microtip) at 40% amplitude. Lysed bacteria were centrifuged at 38465 rcf for 45 min at 4°C. Cleared supernatant was incubated with 5 mL of Glutathione resin (Thermo #16101 LOT#UD285112) with rotation at 4°C for 16 h. Bound protein was washed 3× with 10 mL cold 1× PBS and eluted twice with 10 mL of 25 mM HEPES pH 7.5, 10 mM L-glutathione (Sigma), and 100 mM NaCl. Protein was concentrated by centrifugation at 1,500 rpm on a Sorvall Legend X1 centrifuge in Amicon Ultra-15 centrifugal filter units (UFC#903024). Protein was resuspended in 25 mM HEPES pH 7.5 and 100 mM NaCl and concentrated by centrifugation as above three times. Protein was quantified by absorbance measurement at 280 nm divided by the computed extinction coefficient (ExPASy) (Gasteiger et al., 2003) of the GST-tagged protein domain.

### Kme-OPL Reader Assay

Kme-OPL pools and sets were synthesized by PepScan as C-terminal PEG-biotin conjugates. Binding reactions were performed in 384 deep-well plates (Axygen #P-384-240SQ-C-S). The general procedure per reaction well was as follows. First, 2 μL streptavidin magnetic bead slurry (Pierce, #88817) was washed with Buffer 1 (100 mM NaCl, 25 mM HEPES, pH 7.5, 0.5% BSA (w/v), 0.1% NP-40). Then, 2 μg of peptide pool or set in water was added to washed beads and incubated for 30 min. Reactions were then collected by centrifugation at 2,000 rpm on a Sorvall Legend X1 centrifuge for 2 min prior to being placed on a plate magnet (Alpaqua A001222 LOT#1442). Solution was aspirated and beads were resuspended in 100 μL Buffer 1. These four preceding steps comprised one wash. A second wash was performed, and beads were resuspended in 100 μL GST tagged protein at 125 pmol per well in Buffer 2 (200 mM NaCl, 25 mM HEPES, pH 7.5, 0.5% BSA (w/v), 0.1% NP-40). Following a 30 min incubation, the well was washed 2× and beads were resuspended in 100 μL of a 1:4,000 dilution of primary anti-GST antibody (Sigma #7781) in Buffer 1. Following another 30 min incubation, the well was washed 2× and beads were resuspended in 100 μL of a 1:5,000 dilution of secondary anti-rabbit Alexafluor 488 (Invitrogen #A11034) for 30 min in Buffer 1. The well was again washed 2×, and beads were resuspended in 60 μL of Buffer 1. 40 μL was then transferred to a black 384-well plate (Corning #3575), and fluorescence intensity (485 ± 10 nm excitation filter and 528 ± 10 nm emission filter) was measured with a Synergy HT plate reader (Biotek). All steps were performed at room temperature, as cold incubations did not increase signal (data not shown). All incubation steps after bead resuspensions were performed with the plate on a shaker. For full library screens, peptide-bound beads in Buffer 1 were kept at 4°C for no more than 1 week. The INTEGRA assist plus pipetting robot was used for dispensing all buffers, protein, and antibodies as well as for washing steps. The primary anti-GST antibody alone gave appreciable, position-specific signal on Kme-OPL (Supplementary Figure S2). Therefore, all Kme-OPL reader profiles were performed in parallel with GST control reactions that were subtracted from signals obtained with GST-tagged readers.

### Fluorescence Polarization Assay

Peptides functionalized with N-terminal 5-carboxyfluorescin (FAM) were synthesized by Genscript. All 7-mer motifs were synthesized with flanking glycines to mimic the Kme-OPL design. Binding assays were done in black 384 well plates (Corning #3575). Protein was serially diluted with 10 nM FAM peptide in FP assay buffer (25 mM HEPES pH 7.5, 100 mM NaCl, 0.05% NP-40). Polarization was measured on a Synergy Neo fluorescence plate reader (Biotek) with a 485 ± 10 nm excitation filter and a 528 ± 10 nm emission filter. Measurements were scaled to the last dilution of protein with a requested polarization of 20 milli-polarization units (mP). Anisotropy units (A) were calculated using the equation A = (2P)/(3-P). Dissociation constants were determined by non-linear regression analysis of anisotropy curves by specific binding with Hill slope in GraphPad version 8.3.0.

### Histone Peptide Microarrays

Peptide microarrays were fabricated using an Aushon 2470 microarrayer and used as described (Cornett et al., 2017) with the following modifications. Protein and antibody hybridization steps were performed in buffer containing 25 mM HEPES pH 7.5, 100 mM NaCl, 0.5% BSA (w/v), and 0.1% NP-40. Slides were washed 3 × 5 min in PBS supplemented with 0.1% Tween-20 between each hybridization step. Antibodies used were primary anti-GST (Sigma #7781, 1:2,000 dilution) and an AlexaFluor 647-labeled secondary antibody (Life Technologies A-21245, 1:5,000 dilution). Arrays were scanned using an Innopsys InnoScan 110AL microarray scanner and analyzed using ArrayNinja (Dickson et al., 2016). Full lists of peptides queried by array analysis are in Supplementary Table S2.

### Biotinylated Peptide Pulldowns

Biotinylated peptides were synthesized by the High Throughput Peptide Synthesis and Array Core Facility at UNC Chapel Hill. HEK293 cells were lysed in CSK Buffer (10 mM Pipes pH 7.0, 300 mM sucrose, 100 mM NaCl, 3 mM MgCl_2_) supplemented with 0.1% Triton X-100, Roche Complete EDTA-free protease inhibitor tablet (#11 873 580 001), and Sigma phosphatase inhibitor cocktail 3 (#P0044) for 30 min on ice. Lysates were pre-cleared with 200 μL streptavidin magnetic bead slurry (Pierce #88817) with rotation at room temperature for 30 min. 25 μL bead slurry was washed in buffer containing 25 mM HEPES pH 7.5, 100 mM NaCl, 0.5% BSA (w/v), and 0.1% NP-40 and were then complexed with 50 μg biotinylated peptide for 1 h at room temperature. 50 μg of pre-cleared lysate was added to beads conjugated with peptide and volume was brought up to 500 μL with pulldown buffer. Following a 4-h incubation at 4°C with rotation, beads were washed 3 × 5 min with 500 μL of wash buffer containing 25 mM HEPES, pH 7.5, 200 mM NaCl, 0.5% BSA (w/v), and 0.1% NP-40. Peptides and protein were eluted in 25 μL of 1× SDS loading dye by heating at 95°C for 5 min prior to loading on a 7% polyacrylamide gel for SDS-PAGE. Transfer to PVDF membrane was performed at 45 mA for 90 min using a Hoefer TE77X semi-dry transfer unit. Membranes were blocked with 5% BSA in PBS-T for 15 min prior to incubation with a 1:1,000 dilution of anti-CDYL2 antibody (ab183854 LOT:GR240986-6) in blocking buffer overnight at 4°C with rotation. Membranes were washed 3 × 5 min with 1× PBS-T and incubated in a 1:10,000 dilution of HRP-conjugated secondary antibody (GE #NA934V) in blocking buffer. Membranes were exposed to ECL substrate (Pierce 32209) following 3 × 5 min washes with 1× PBS-T and imaged with Kodak × omat. Images were quantified with ImageJ Version 1.52.

## Results

### A Kme Reader Assay Querying Kme-OPL

The Kme-OPL reader platform is a plate-based magnetic bead pulldown assay read out by fluorescence intensity (Figure 1C). The library is oriented around a central lysine, which can have one of four possible methyl orders (Kme0, Kme1, Kme2, or Kme3) (Figure 1A). Within each methyl order, the library is organized into 114 Kme-OPL sets, where each set has one amino acid fixed in one position. All other positions contain 19 amino acids in an equimolar, degenerate mix. Cysteine is excluded due to incompatibility with the synthetic approach. The peptides are biotinylated, which allows for binding to streptavidin magnetic beads. The beads and Kme-OPL sets are first complexed, and then a recombinant GST-tagged Kme reader is added (Figure 1C). Next, a primary GST antibody followed by a secondary antibody conjugated to a fluorophore are added. Binding is read out by fluorescence intensity measurements. The optimization of several assay components is present in Supplementary Figure S1 and further detailed in section “Materials and Methods.”

### Methyl Order Preferences for Histone Kme Readers Are Recapitulated With Kme-OPL

**FIGURE 2:**
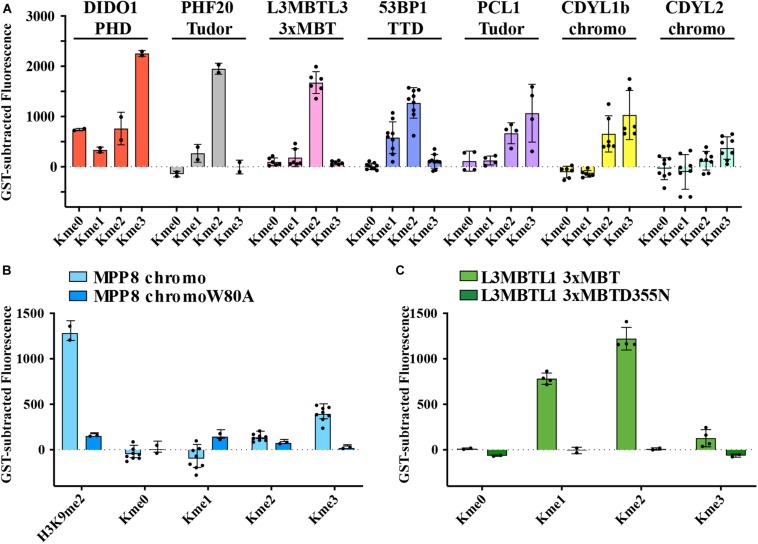
Preferred methyl orders for histone Kme readers are recapitulated with Kme-OPL. **(A–C)** GST-subtracted fluorescent signals from Kme-OPL pools reacted with the indicated wild-type or mutant Kme reader domains. Error bars are SD from replicate binding reactions (black dots). A biotinylated H3_(__1__–__20__)_K9me2 peptide was included in **B**.

Kme readers have been reported to prefer the same lysine methyl order on histone and non-histone proteins (Cui et al., 2012; Liu et al., 2013; Ferry et al., 2017). We first tested whether the Kme-OPL platform could detect Kme reader methyl order preference. To measure preferred methyl order, we synthesized Kme-OPL pools, where all peptides sets with the same methyl order are combined into a single pulldown reaction. We queried nine reader domains known to bind histone Kmes (Table 1, Supplementary Figures S3A, S4A and Figure 5C). Each measurement is reported as a GST subtracted value (Figure 2). Most values had a simultaneous GST measurement subtracted. A small subset of experiments had high GST signals for unknown reason (Supplementary Figure S2). For experiments without a simultaneous GST measurement, we inferred whether low or high background signal should be subtracted based on the signal from the Kme0-OPL pool. In each case, binding to Kme-OPL pools was consistent with reported histone methyl order preferences (Figure 2A). To further test if signal was dependent on Kme binding, we assayed mutant forms of MPP8 chromo and L3MBTL1 3xMBT which had single amino acid substitutions known to disrupt their interactions with Kmes (Li et al., 2007; Chang et al., 2011). In both mutants, signal intensities were reduced to GST background levels (Figures 2B,C). We note variability in max signals with Kme-OPL pools, which we interpret as either weak overall affinity or high affinity to a limited set of peptides. Later, we resolve this mixed interpretation.

**TABLE 1:** Reported histone interactions for Kme readers queried in Figure 2.

**Protein domain**	**Associated histone mark**
MPP8 chromo	H3K9me2/3 (Kokura et al., 2010)
L3MBTL1 3xMBT	H1bK26me1/2 (Trojer et al., 2007) H4K20me1/2 (Min et al., 2007)
DIDO1 PHD	H3K4me3 (Gatchalian et al., 2016)
PHF20 Tudor	H3K4me2 (Klein et al., 2016) H4K20me2 (Klein et al., 2016)
L3MBTL3 3xMBT	Many Kme2 (Nady et al., 2012)
53BP1 TTD	H4K20me1/2 (Botuyan et al., 2006; Hartlerode et al., 2012) H3K18me2 (Shanle et al., 2017) H3K36me2 (Tong et al., 2015)
PCL1 Tudor	H3K36me3 (Cai et al., 2013)
CDYL1b chromo	H3K9me2/3 (Franz et al., 2009; Escamilla-Del-Arenal et al., 2013) H3K27me3 (Vermeulen et al., 2010)
CDYL2 chromo	H3K9me3 (Fischle et al., 2008) H3K27me3 (Fischle et al., 2008)

### Kme-OPL Reports on Sequence Determinants of Kme Reader Specificity

We next used the Kme-OPL platform to determine how the amino acid sequence surrounding the Kme modulated reader binding (Kme-OPL profile). We used this sequence data in conjunction with Lowest Bin (LoB) scoring (Cornett et al., 2018) to predict where these readers may bind in the proteome (Figure 1B). For these studies, MPP8 and CDYL2 chromodomains were chosen because aspects of their amino acid binding preferences are reported elsewhere (Li et al., 2011; Barnash et al., 2016), and these data were consistent with Kme-OPL profiles generated with these readers.

**FIGURE 3:**
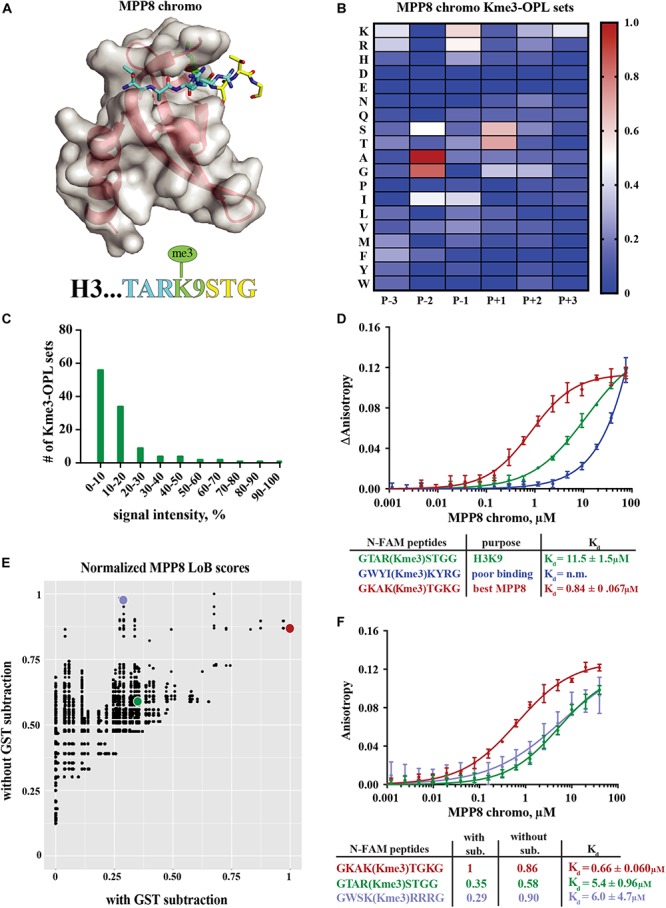
Binding determinants of MPP8 chromo and validation of Lowest Bin (LoB) scoring. **(A)** MPP8 chromo co-structure with an H3K9me3 peptide (PDB:3QO2). **(B)** MPP8 chromo Kme3-OPL profile. Each Kme3-OPL set is shown in the heatmap as an average of 4 replicate GST subtracted fluorescence measurements, and data is scaled from 0 (no signal, blue) to 1 (highest average signal, red). **(C)** MPP8 chromo specificity profile. Specificity is graphed as the number of Kme3-OPL sets with a given signal intensity range. **(D)** MPP8 chromo fluorescence polarization. Data points are plotted as an average of 4 measurements from 2 independent experiments. Error is SD **(E)** Scatterplot of normalized MPP8 LoB scores from data with GST subtraction vs without GST subtraction. Red, green, and blue points correspond to same colored peptides as in **F**. **(F)** Fluorescence polarization of MPP8 chromo. Data points are plotted as an average of 5 replicate measurements from 2 independent experiments. Error is SD.

The MPP8 chromodomain structure is a three stranded antiparallel β sheet with a C terminal α helix (Li et al., 2011). In the H3K9 tail sequence, Q5, T6, and A7 interact with the residues V58, F59, E60 and V61 in MPP8 and induce creation of another β strand (β1), forming a β hairpin (Li et al., 2011; Figure 3A). H3S10 forms a non-backbone hydrogen bond with MPP8 residue E91. For residues succeeding S10, no interactions are observed. Because conformational induction of the β hairpin is essential for Kme binding, a specific sequence context that will recapitulate these contacts is necessary. These data suggest the MPP8 Kme3-OPL pool signal is low (Figure 2A) because a limited number of pool peptides can induce this conformational change.

The MPP8 chromo Kme-OPL profile had clear position preferences that aligned with the crystal structure (Figure 3B and Supplementary Figures S3B,C). As with Kme-OPL pools measurements, Kme-OPL profiles were GST background subtracted (Supplementary Figure S2B). In the MPP8 Kme3-OPL profile, P-3 slightly preferred basic or aromatic amino acids. P-2 strongly favored A/G. The preference of P-2 toward smaller amino acids is likely due to this position’s location inside the β hairpin. P-1, a position that performs Van der Waals interactions (Li et al., 2011), preferred K/R/I. The same P-2 and P-1 preferences have been reported for the chromodomains of CBX proteins (Kaustov et al., 2011). CBX2/3/5/6/7/8 all had P-2 in a small hydrophobic pocket that could only fit alanine or smaller residues, and CBX7 preferred P-1 R/I/L/F/Y/V. The conserved preferences across chromodomains further support our results for MPP8. Continuing with the MPP8 profile, P+1 strongly favored S/T, likely because of the ability of these amino acids to form a non-backbone hydrogen bond. MPP8 chromo did not have a P+2 amino acid preference, which is in accordance with no contacts being made in this position in the H3 co-structure (Figure 3A). Although there are also no contacts being made in P+3 in the co-structure, our assay revealed a P+3 preference for lysine (Figure 3B). Figure 3C shows a histogram of MPP8 Kme3-OPL set signals and provides an easy way of determining if a protein is sequence specific. We consider MPP8 chromo to be sequence specific because only a few Kme3-OPL sets had high signals while the majority were shifted toward lower values. Collectively, the MPP8 chromo Kme3-OPL profile showed preference for several amino acids that would be predicted from the structure of MPP8 chromo bound to H3K9me3.

We next asked whether we could use the MPP8 Kme3-OPL profile to predict an optimal binding sequence. We predicted the best binding sequence, KAK(Kme3)TGK, by choosing the Kme3-OPL set with the highest signal in each position. We compared this sequence to the sequence surrounding H3K9me3, TAR(Kme3)STG, and also to a predicted poor binding sequence, WYI(Kme3)KYR, chosen by picking Kme3-OPL sets with low signals in each position. We measured the *K*_*d*_ of the MPP8 chromo interaction with each peptide using fluorescence polarization (FP). The predicted poor binding peptide had a *K*_*d*_ that was too weak to be determined (Figure 3D). The best predicted peptide had a *K*_*d*_ of 0.84 +0.067 μM, binding ten-fold tighter than the H3K9me3 peptide. Since this tight-binding peptide was present in the Kme3-OPL pools, the low overall signal of MPP8 toward the Kme3-OPL pool was unlikely due to low affinity to the entire pool (Figure 2). Rather, MPP8 bound strongly to only a few sequences that were diluted in the pools, resulting in a lower Kme-OPL pool signal.

In order to relate Kme-OPL profiles to the human proteome, we used our previously developed LoB scoring function (Cornett et al., 2018). LoB scoring ranks all lysine centered seven-mers in the proteome from most to least likely to bind to a given reader. This was original developed to identify lysine methyltransferase substrates but can also be applied to identify Kme reader interactions. LoB scoring minimizes false positives by having the lowest Kme-OPL set dictate the score. All LoB scoring is deposited at https://github.com/ariana-kupai/LoB_scores as GST background subtracted values. The reasons for GST subtraction are described below.

LoB scores generated from MPP8 chromo screening were plotted with and without GST subtraction and normalized to their respective highest score to facilitate comparisons (Figure 3E). Each dot on the scatterplot represents a lysine-centered seven-mer in the proteome. Three peptides were chosen with ranging LoB scores (TAR(Kme3)STG, KAK(Kme3)TGK, and WSK(Kme3)RRR) for comparison in FP binding assays (Figure 3F). With MPP8 chromo, TAR(Kme3)STG had a *K*_*d*_ of 5.4 +0.96 μM, KAK(Kme3)TGK had a *K*_*d*_ of 0.66 +0.060 μM, and the *K*_*d*_ for WSK(Kme3)RRR was 6.0 +4.7 μM. The FP binding results led us to conclude that background subtracted LoB scores were more reflective of *in vitro* binding constants. Consequently, we have reported LoB scoring only on GST subtracted Kme-OPL profiles, which should help further reduce selection of false positives for downstream studies. Of note, the preferred sequence for MPP8 chromo, KAKKTGK, mapped to Calcium permeable stress-gated cation channel 1 (CSC1) in the human proteome. However, because CSC1 localizes to the plasma membrane and MPP8 is found in the nucleus, this interaction is not likely to be physiologically relevant.

**FIGURE 4:**
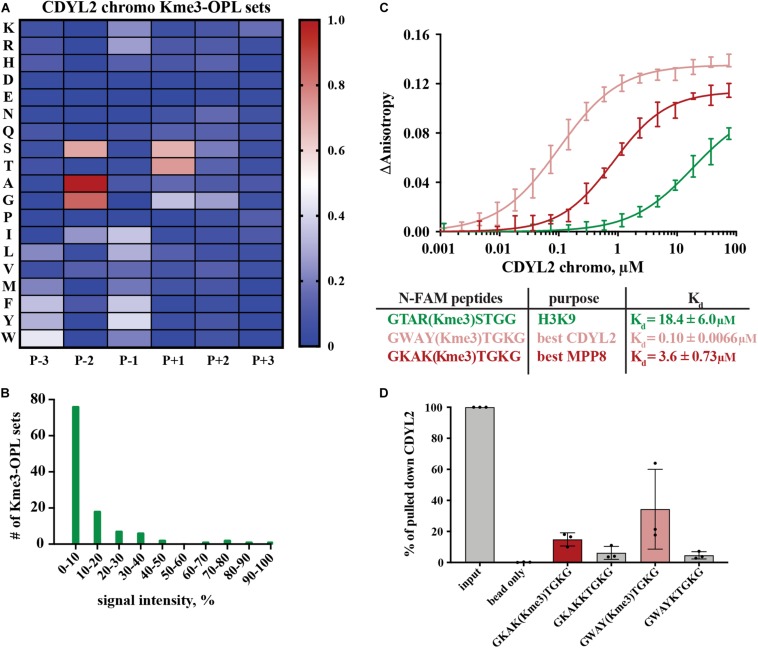
Binding determinants of CDYL2 chromo. **(A)** CDYL2 chromo Kme3-OPL profile. Each Kme3-OPL set is shown in the heatmap as an average of 4 GST subtracted fluorescence measurements from 2 independent binding reactions. Data is scaled as in Figure 3B. **(B)** CDYL2 chromo specificity profile. **(C)** CDYL2 chromo fluorescence polarization. Data points are plotted as an average of 4 measurements from 2 independent experiments. Error is SD **(D)** Quantification of CDYL2 signal from western blots of biotinylated-peptide pulldown from HEK293 cell lysates (see also Supplementary Figure S5C). Quantified signal from each lane was normalized to input signal from the same blot. Error bars are SD.

MPP8 and CDYL2 chromodomains have similar structures and both recognize H3K9me2/me3 (Fischle et al., 2008; Supplementary Figures S3A, S4A). We next sought to compare Kme-OPL profiles of these closely related proteins. Certain positional binding preferences were conserved between MPP8 and CDYL2 chromodomains, as anticipated from their recognition of the same histone Kme. In both Kme-OPL profiles, P-2 was the most selective position, favoring A/G (Figures 3B, 4A and Supplementary Figure S4B). Both proteins also favored P+1 S/T. CDYL2 had a more specific profile than MPP8 (Figures 3C, 4B). MPP8 signals tapered off while CDYL2 signals had a bimodal distribution, signifying more amino acids promoted or inhibited binding.

Peptide scaffolds are the basis for some Kme reader antagonists (James et al., 2013; Simhadri et al., 2014; Stuckey et al., 2016). Notably, the Kme-OPL profile of CDYL2 highlighted similar characteristics of amino acids in the CDYL2 Kme peptidomimetic inhibitor, UNC4991 (Barnash et al., 2016). UNC4991 has P-3 F, consistent with the preference the Kme3-OPL profile showed for P-3 non-polar aromatic residues (Figure 4A). UNC4991 has P-2 A and our assay showed preference for P-2 A/G. UNC4991 has P-1 F and our assay showed preference for P-1 non-polar residues. UNC4991 has P+1 T and our assay had P+1 preference for S/T. UNC4491 lacks P+2 and P+3 residues, consistent with the lack of amino acid preference in these positions on Kme-OPL. Using the Kme-OPL assay, we converged upon the same characteristics of amino acids that promoted peptide binding to CDYL2. This further validates our CDYL2 chromo Kme3-OPL profile and demonstrates the potential utility of the Kme-OPL platform for designing peptide-based inhibitors.

We next used the Kme3-OPL profile for CDYL2 to predict an optimal binding sequence. The predicted best binding sequence was WAY(Kme3)TGK, which had a *K*_*d*_ of 0.10 ± 0.0066 μM as measured by FP. This peptide, which does not map to any human protein, bound 100-fold tighter than the H3K9me3 peptide (Figure 4C). We also measured the *K*_*d*_ of CDYL2 chromo with KAK(Kme3)TGK, the best MPP8 peptide. This interaction had a *K*_*d*_ of 3.6 ± 0.73 μM. We functionalized these sequences (methylated and unmethylated) with biotin and performed peptide pulldowns for CDYL2 from HEK293 cell lysates. A lysate titration and western blot images from three independent experiments are in Supplementary Figure S5. In three replicate experiments, the tri-methylated sequences pulled down more CDYL2 than the unmethylated sequences. In one of the replicates, WAY(Kme3)TGK pulled down more CDYL2 than KAK(Kme3)TGK, consistent with the *in vitro* observation (Figure 4D). These pulldowns were performed with 3 independent preparations of cell lysate. Therefore, variables like cell cycle distribution of the bulk population, protein posttranslational modifications, and abundance of competitively binding proteins cannot be ruled out as variables impacting the reproducibility of these and other pulldowns from cell extracts.

We also measured the *K*_*d*_ of MPP8 chromo with WAY(Kme3)TGK, the best CDYL2 peptide (Supplementary Figure S4C). The *K*_*d*_s of MPP8 chromo with the best MPP8 peptide and the best CDYL2 peptide were very similar (Figure 3D). Comparatively, MPP8 is a less specific reader than CDYL2. P-2 A and P(+1 T may have greater impact than other positions for driving interactions with MPP8, making the MPP8 and CDYL2 best peptides equally strong binding sequences. Collectively, these results show the Kme-OPL platform can be used to identify preferred amino acid sequences for very specific reader domains.

### Kme-OPL Recognized Promiscuity in Kme Reader Binding

**FIGURE 5:**
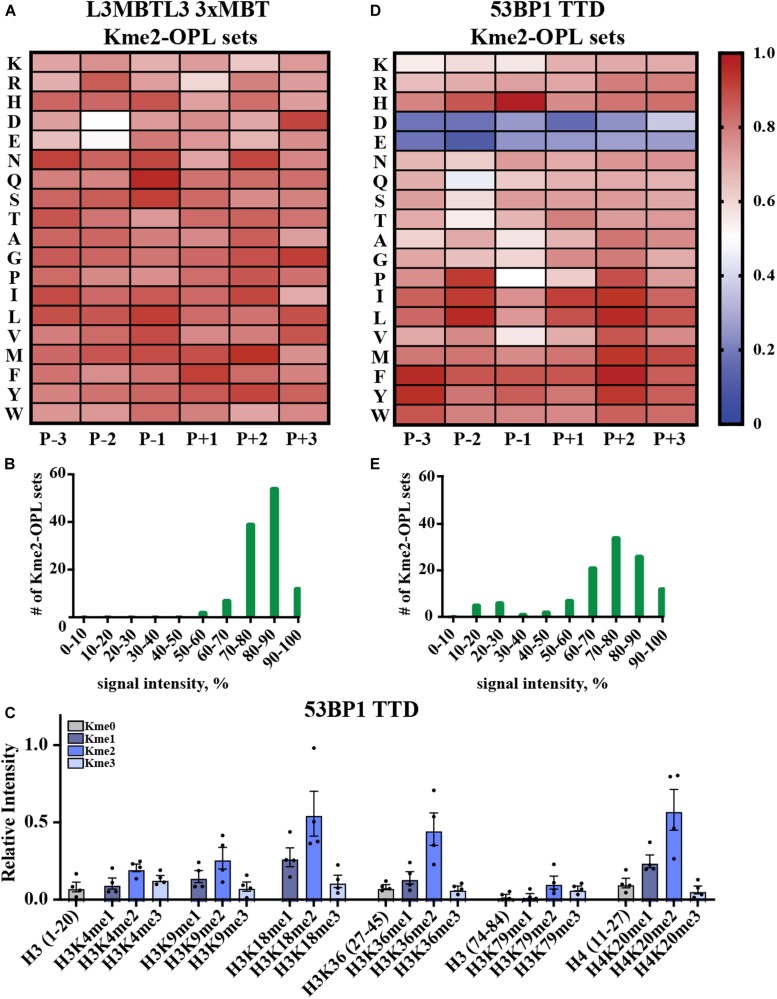
Promiscuity in Kme binding is recognized by Kme-OPL. **(A)** L3MBTL3 3xMBT Kme2-OPL profile. Two independent binding measurements with 2–3 technical replicate measures for each Kme2-OPL set were averaged. Data was GST background subtracted and normalized as in Figure 3B. **(B)** L3MBTL3 3xMBT specificity profile. **(C)** 53BP1 TTD histone peptide microarray data. Data points were normalized to the highest peptide signal, and error is SEM **(D)** 53BP1 TTD Kme2-OPL profile. Two independent binding measurements with 2 technical replicate measures for each Kme2-OPL set were averaged. Data was GST subtracted and normalized as in Figure 3B. **(E)** 53BP1 TTD specificity profile.

Kme-OPL reported amino acid binding preferences for sequence specific Kme readers, so we next sought to determine what Kme-OPL would report for non-specific readers. L3MBTL3 is a promiscuous Kme2 reader whose 3xMBT domain binds to many Kme2 histone contexts *in vitro* (Nady et al., 2012). L3MBTL3 was also classified as a promiscuous Kme2 reader by our assay. The Kme2-OPL profile for L3MBTL3 3xMBT tolerated all residues (Figure 5A), and most Kme-OPL sets had high signals (Supplementary Figures S6A,B and Figure 5B). These results are consistent with previous studies that show surrounding amino acids do not impact L3MBTL3’s mechanism of Kme recognition (Li et al., 2007) or potency of the L3MBTL3 peptidomimetic inhibitor UNC1215 (James et al., 2013); both of which lack protein-peptide contacts outside of the Kme (Supplementary Figure S6C). Also consistent with a previous report (Nady et al., 2012), the lowest signals in the L3MBTL3 Kme2-OPL profile belonged to acidic residues in the P-2 position (Figure 5A).

Another promiscuous Kme reader is 53BP1. 53BP1 TTD is reported to bind p53K370me2, p53K382me2 (Roy et al., 2010), H4K20me1/2 (Botuyan et al., 2006; Hartlerode et al., 2012), H3K18me2 (Shanle et al., 2017) and H3K36me2 (Tong et al., 2015) (Table 1 and Supplementary Figure S7A). Our peptide microarray data confirmed the ability of 53BP1 TTD to recognize these histone Kmes (Figure 5C). The sequences surrounding these Kme sites are not conserved (Supplementary Figure S7B), signifying 53BP1 TTD is a non-specific Kme reader. Kme-OPL pool screening was consistent with prior reports showing 53BP1 TTD preferred Kme2 (Figure 2A). The Kme2-OPL profile showed 53BP1 TTD interactions were largely non-specific, binding to Kme2 in almost all sequences, with the exception of acidic residues (Figures 5D,E and Supplementary Figures S7C,D).

From our collective analyses of Kme readers, it was apparent that sequence-specific Kme readers had lower Kme-OPL pool signals than non-specific readers (Figure 2). The Kme3-OPL pool signal average for CDYL2, the most sequence-specific reader in our screen, was only ∼400 RFU. The low signal was explained by CDYL2 chromo tolerating few residues (Figure 4B), leaving CDYL2 only able to bind to a small number of peptides in each pool. The opposite was shown for L3MBTL3 3xMBT, the most sequence promiscuous reader. L3MBTL3 3xMBT had a high signal, ∼1,600 RFU, for the Kme2-OPL pool. L3MBTL3 bound to di-methylated peptides regardless of amino acid sequence (Figure 5B). Consequently, L3MBTL3 was able to bind to most peptides in the Kme2 pool, resulting in a high signal.

### Kme-OPL Revealed Few Sequence Determinants for Histone Kme-Specific Antibody Target Recognition

**FIGURE 6:**
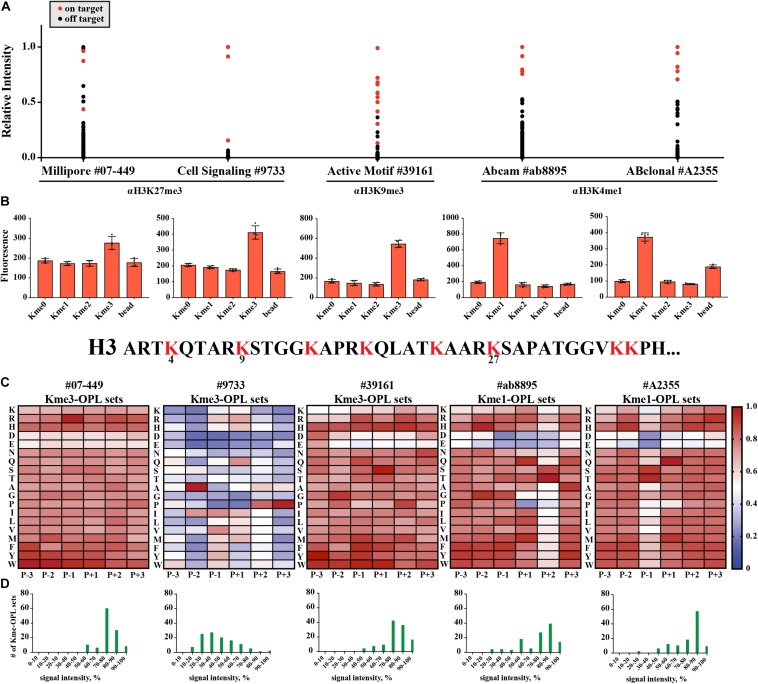
Kme-OPL reveals few sequence drivers for Kme-specific antibody target recognition. **(A)** Histone peptide microarray data. Signals were normalized to the highest peptide average per subarray. On target is defined as a peptide that contains the intended methyllysine mark. **(B)** Kme-OPL pools. Bar graphs are an average of 4 replicate measurements. Error is SD **(C)** Kme-OPL profiles. Profiles are an average of 4 fluorescent binding measurements. ab8895 is 2 individual experiments while all others are replicates from one experiment. Data was normalized as in Figure 3B. **(D)** Histone Kme antibody specificity profiles. Millipore 07-449 LOT: 2455635 dilution 1:5,000, CST 9733 LOT:14 dilution 1:5,000. Active Motif #39161 LOT: 14418003 dilution 1:5,000. ab8895 LOT: GR141677-4 dilution 1:10,000. ABclonal A2355 combination of LOT: 2200170102 and 2200170202 dilution 1:5,000.

We wondered what our assay would report as the sequence determinants of histone Kme-specific antibodies, as antibodies are not generally characterized in an unbiased way with regard to the sequences they are presented. We assayed five antibodies: Millipore #07-449, anti-H3K27me3; Cell Signaling Technologies #9733, anti-H3K27me3; Active Motif #39161, anti-H3K9me3; Abcam #ab8895, anti-H3K4me1; and ABClonal #A2355, anti-H3K4me1. First, we used histone peptide microarrays to establish whether an antibody was specific for its target histone Kme (Figure 6A). Next, we determined methyl order specificity of each antibody by proxy of the Kme-OPL pools (Figure 6B). Finally, we queried sequence preferences by assaying each antibody on Kme-OPL sets of the preferred methyl order (Figure 6C and Supplementary Figure S8). Each antibody was specific for its intended methyl order. Surprisingly, antibodies were sequence tolerant (Figure 6D), and the determinants of antibody sequence specificity were dictated by, at most, two amino acid positions (Figure 6C). Millipore #07-449 lacked target specificity on both peptide microarray and Kme3-OPL sets. Cell Signaling Technologies #9733 was the most specific antibody on microarray, but Kme3-OPL profiling showed the antibody’s recognition of H3K27 likely came from selectivity in only two positions, P-2 A and P+3 P. Active Motif #39161 was not specific on microarray and did not have a specific Kme3-OPL profile. The two H3K4me1 antibodies, Abcam #ab8895 and ABClonal #A2355, had similar microarray and Kme1-OPL readouts. Both antibodies recognized H3K4me1 on microarray and were sequence selective in one position in the Kme1-OPL. #ab8895 was specific for P+2 S/T, and #A2355 was specific for P-1 S/T. Both antibodies slightly preferred P+1 Q, and the combination of P+1 Q and P+2 S/T or P-1 S/T is unique to H3K4, the intended antibody target. The most specific antibodies, #9733, #ab8895 and #A2355, recognized their target Kme by only two selective positions.

LoB scoring of histone antibody Kme-OPL profiles revealed that, from a strictly sequence selectivity perspective, these antibodies were unlikely to recognize their intended target. For example, in the LoB scoring of ab8895 anti-H3K4me1, 364 lysine-centered seven-mer motifs in the proteome had a LoB score equal to or higher than that of H3K4. ab8895 is the most cited H3K4me1 antibody (Shah et al., 2018). Undoubtedly, ab8895 recognizes H3K4me1, but we cannot explain this solely by high-affinity interactions, as we predict 364 sequences to bind equally or better than H3K4. Likely, the community does not robustly detect off target proteins in techniques like western blot (He et al., 2019) or ChIP (Mohaghegh et al., 2019) because histones are so much more abundant, as reported by Wisniewski et al. (2014). ab8895 anti-H3K4me1 LoB scores ranged from 0 to 801.5. The highest ab8895 LoB scoring protein present in Wisniewski’s mass spectrometry data was Sideroflexin-4, which had a LoB score of 784.25. Comparatively, H3K4 had a LoB score of 738.5. Averaging three measurements and reporting the standard deviation, the copy number of Sideroflexin-4 was 29 ± 6.5 × 10^3^ particles/cell, and the copy number of H3.1 was 33 ± 4.3 × 10^6^ particles/cell; H3.1 was 1,100 times more abundant than Sideroflexin-4. Since sequences with higher LoB scores exhibited higher binding affinity (Figure 3F), Sideroflexin-4 likely binds to ab8895 with higher or at least the same affinity as H3K4. However, binding affinity does not equate to antibody recognition when amounts of targets are so different. High abundance of histones has worked in our favor in the chromatin field. Conversely, studying methylated proteins that are not histones using antibodies will be challenging. Even if an antibody is specific for a protein, the abundance of histones or other competing proteins may obscure detection.

## Discussion

Here we report on the development of a Kme-OPL platform that is able to capture the optimal binding sequence P-3/+3 for Kme reader domains and Kme antibodies. Kme-OPL profiles were validated with structural and quantitative binding data, corroborating that Kme-OPL profiles were accurate and could be used to predict optimal binding sequences. Additionally, LoB scoring utilized the surrounding amino acid sequence information to relate Kme-OPL findings to the proteome, toward the goal of identifying potential protein-protein interactions. In the future, this assay can be used for *de novo* characterization of putative Kme readers with no known activity and in drug discovery pipelines, for the identification of high affinity ligands for screening assays, and for the design of peptidomimetic Kme reader antagonists.

The Kme-OPL approach for identifying Kme driven interactions is complimentary to other available tools such as mass spectrometry and SPOT array. When a Kme reader is pulled down from cells and analyzed by mass spectrometry, the nature of identified interactions is unknown. LoB scoring of a Kme reader can be used to prioritize potential direct binding partners. This assay can also inform on optimal protein binding sequences and can be used in SPOT array construction, which relies on prior knowledge of protein binding motifs.

The Kme-OPL reader assay has a few limitations. One limitation of the assay is the use of Kme-OPL pools. Currently, we use the Kme-OPL pools to determine a preferred methyl order for a Kme reader, and the Kme-OPL pool with the highest signal dictates the methyl order used for measurements with Kme-OPL sets. A protein that selectively binds to only few residues may give no detectable signal in Kme-OPL pool screening and would not be continued in our current workflow. The Kme-OPL pool step therefore may report false negatives for highly sequence-specific Kme readers. Another limitation of this assay is the peptide library being constructed with residues P-3/+3 of the Kme. Although the best MPP8 chromo peptide had a *K*_*d*_ of 0.84 ± 0.067 μM, making it ten-fold tighter than the H3K9me3 peptide of the same length (Figure 3C), the H3K9me3 (1-20) peptide had a *K*_*d*_ of 0.23 ± 0.03 μM (Rothbart et al., 2012). For the CDYL2 inhibitor, specific amino acids in the P-4 position also increased binding affinity (Barnash et al., 2016). Conversely, residues P+2 and P+3 were not informative for CDYL2 binding and were not included in inhibitor design. Here, we inform on residues most likely to interact with a Kme reader, but P-3/+3 of the Kme will not be the binding footprint for every protein or antibody.

## Data Availability Statement

Datasets generated for this study can be found at GitHub (https://github.com/ariana-kupai/LoB_scores) and the Histone Antibody Specificity Database (www.histoneantibodies.com).

## Author Contributions

AK designed and performed experiments and analyzed data. RV designed experiments and analyzed data. BD developed code, designed experiments, and analyzed data. SR designed and performed experiments and analyzed data. All authors contributed to the writing of the manuscript.

## Conflict of Interest

The authors declare that the research was conducted in the absence of any commercial or financial relationships that could be construed as a potential conflict of interest.

## Abbreviations

BPTF-BRD-PHD: Bromodomain Plant Homeodomain Finger Transcription Factor Bromodomain-Plant Homeodomain
CBX: polycomb chromobox
CDYL1b chromo: Chromodomain Y-like protein 1b chromodomain
CDYL2 chromo: Chromodomain Y-like protein 2 chromodomain
DIDO1 PHD: Death-Inducer Obliterator 1 Plant Homeodomain
GST: Glutathione S Transferase
JMJD2a TTD: Jumonji domain- containing protein 2a Tandem Tudor domain
Kme: methyllysine
Kme-OPL: methyllysine-oriented peptide library
L3MBTL1 3xMBT: Lethal 3 Malignant Brain Tumor-like protein 1 3 Malignant Brain Tumor domains
L3MBTL3 3xMBT: Lethal 3 malignant brain tumor-like protein 3 3 malignant brain tumor domains
MPP8 chromo: M Phase Phosphoprotein 8 chromodomain
PCL1 Tudor: Polycomb-like Protein 1
PHF20 Tudor: Plant Homeodomain Finger Protein 20
RFU: relative fluorescence units
UHRF1 TTD: Ubiquitin-like containing PHD and RING finger domains 1 Tandem Tudor domain
53BP1 TTD: p53 Binding Protein 1 Tandem Tudor domain.
