# Supplementary material for: A Degenerate Peptide Library Approach to Reveal Sequence Determinants of Methyllysine-Driven Protein Interactions
Source: Front Cell Dev Biol. 2020 Apr 9;8:241. doi: 10.3389/fcell.2020.00241 (PMC7160673; doi:10.3389/fcell.2020.00241)
Supplement: Supplementary file 1 [file Data_Sheet_1.pdf]

Supplementary Material

Supplementary Table 1. N-terminal GST-fusion plasmids used in this study

Protein Domain	Amino Acids	Source	Reference
53BP1 TTD	1459-1635	Gift from M. Bedford	(Shanle et al., 2017)
BPTF PHD-BRD	2722-2890	Gift from A. Ruthenburg	(Ruthenburg et al., 2011)
CDYL1b chromo	1-78	Gift from M. Bedford	(Shanle et al., 2017)
CDYL2 chromo	1-75	Gift from M. Bedford	(Shanle et al., 2017)
DIDO PHD	266-325	Gift from T. Kutateladze	(Gatchalian et al., 2013)
L3MBTL1 3x MBT	190-530	Gift from O. Gozani	(Moore et al., 2013)
L3MBTL1 3x MBT D355N	190-530	Gift from O. Gozani	(Moore et al., 2013)
L3MBTL3 3xMBT	225-555	Gift from M. Bedford	(Shanle et al., 2017)
MPP8 chromo	50-118	Gift from M. Bedford	(Rothbart et al., 2012)
MPP8 chromo W80A	50-118	Made for this study	(Chang et al., 2011)
PCL1 Tudor	29-82	Gift from G. Wang	(Cai et al., 2013)
PHF20 Tudor	58-148	Gift from M. Bedford	(Shanle et al., 2017)
UHRF1 TTD	123-284	Made for this study	This study
UHRF1 TTD-PHD	123-366	Gift from B. Strahl	(Rothbart et al., 2013)

Supplementary Methods

Kme-OPL reader assay optimization

The following section describes experiments performed to optimize various aspects of the Kme-OPL pulldown procedure for Kme readers. We used the TTD-PHD of UHRF1 for these initial studies, which we and others have reported binds to H3K9me2 and H3K9me3 with nM affinity (Arita et al., 2012; Rothbart et al., 2013). First, we considered volume of streptavidin-coated magnetic bead slurry used per reaction. With both an H3K9me2 peptide and the P-1 R Kme2-OPL set, we measured the same signal intensity at 2 µL and 8.3 µL of beads (**Supplementary Figure S1A**). A 2 µL bead slurry has the binding capacity of 2 µg biotinylated peptide, which we determined from the following calculation, would give sufficient library representation. The average weight of one peptide in our library is 1703.5 Da, so 2 µg of peptide averages to 1.17×10^{-9} moles. A 2 µL bead slurry can bind 7×10^{-11} moles of biotinylated peptide. One Kme-OPL set has 2.48×10^{6} sequence combinations. At binding capacity of a 2 µL bead slurry, all sequence combinations are represented 1.7×10^{7} times. One Kme-OPL pool has 282.3×10^{6} sequence combinations, and at binding capacity, all combinations are represented 1.49×10^{5} times.

To minimize non-specific interactions, we next considered NaCl as a variable in Tris pH 7.5 and HEPES pH 7.5 reaction buffers. We again used UHRF1 TTD-PHD and Kme2-OPL sets. Based off the H3K9 sequence, we chose P-1 R to represent a strong interaction, P-1 S an intermediate one, and P-1 E a weak one. Signals in Tris were lower than in HEPES, and for both buffers, signal was decreased in a salt-dependent manner (**Supplementary Figure S1B,C**). From these data, we chose to run all assays in HEPES buffer with 200 mM NaCl.

To understand how protein concentration affected signal, we titrated UHRF1 TTD and BPTF PHD-BRD, H3K9me3 and H3K4me3 readers, respectively (Nady et al., 2011; Rothbart et al., 2012; Ruthenburg et al., 2011), with Kme3-OPL sets P-1 R and P-1 T, expecting each protein to prefer the peptide mimicking its associated lysine in the histone sequence context (**Supplementary Figure S1D,E**). For both proteins, signal increased as a function of protein concentration and began to reach saturation between 125 pmol and 250 pmol. Based on these results, 125 pmol of protein was chosen for this assay.

Finally, we optimized primary and secondary antibody concentrations with MPP8 chromo, an H3K9me2/me3 reader (Kokura et al., 2010), and the Kme3-OPL set P-2 A (**Supplementary Figure S1F**). From this pairwise titration analysis, we chose 1:4000 primary and 1:5000 secondary antibody dilutions.

Supplementary References

Arita, K., Isogai, S., Oda, T., Unoki, M., Sugita, K., Sekiyama, N., Kuwata, K., Hamamoto, R., Tochio, H., Sato, M., *et al.* (2012). Recognition of modification status on a histone H3 tail by linked histone reader modules of the epigenetic regulator UHRF1. Proc Natl Acad Sci U S A *109*, 12950-12955.

Cai, L., Rothbart, S.B., Lu, R., Xu, B., Chen, W.Y., Tripathy, A., Rockowitz, S., Zheng, D., Patel, D.J., Allis, C.D., *et al.* (2013). An H3K36 methylation-engaging Tudor motif of polycomb-like proteins mediates PRC2 complex targeting. Mol Cell *49*, 571-582.

Chang, Y., Horton, J.R., Bedford, M.T., Zhang, X., and Cheng, X. (2011). Structural insights for MPP8 chromodomain interaction with histone H3 lysine 9: potential effect of phosphorylation on methyl-lysine binding. J Mol Biol *408*, 807-814.

Gatchalian, J., Futterer, A., Rothbart, S.B., Tong, Q., Rincon-Arano, H., Sanchez de Diego, A., Groudine, M., Strahl, B.D., Martinez, A.C., van Wely, K.H., *et al.* (2013). Dido3 PHD modulates cell differentiation and division. Cell Rep *4*, 148-158.

Kokura, K., Sun, L., Bedford, M.T., and Fang, J. (2010). Methyl-H3K9-binding protein MPP8 mediates E-cadherin gene silencing and promotes tumour cell motility and invasion. EMBO J *29*, 3673-3687.

Moore, K.E., Carlson, S.M., Camp, N.D., Cheung, P., James, R.G., Chua, K.F., Wolf-Yadlin, A., and Gozani, O. (2013). A general molecular affinity strategy for global detection and proteomic analysis of lysine methylation. Mol Cell *50*, 444-456.

Nady, N., Lemak, A., Walker, J.R., Avvakumov, G.V., Kareta, M.S., Achour, M., Xue, S., Duan, S., Allali-Hassani, A., Zuo, X., *et al.* (2011). Recognition of multivalent histone states associated with heterochromatin by UHRF1 protein. J Biol Chem *286*, 24300-24311.

Rothbart, S.B., Dickson, B.M., Ong, M.S., Krajewski, K., Houliston, S., Kireev, D.B., Arrowsmith, C.H., and Strahl, B.D. (2013). Multivalent histone engagement by the linked tandem Tudor and PHD domains of UHRF1 is required for the epigenetic inheritance of DNA methylation. Genes Dev *27*, 1288-1298.

Rothbart, S.B., Krajewski, K., Nady, N., Tempel, W., Xue, S., Badeaux, A.I., Barsyte-Lovejoy, D., Martinez, J.Y., Bedford, M.T., Fuchs, S.M., *et al.* (2012). Association of UHRF1 with methylated H3K9 directs the maintenance of DNA methylation. Nat Struct Mol Biol *19*, 1155-1160.

Ruthenburg, A.J., Li, H., Milne, T.A., Dewell, S., McGinty, R.K., Yuen, M., Ueberheide, B., Dou, Y., Muir, T.W., Patel, D.J., *et al.* (2011). Recognition of a mononucleosomal histone modification pattern by BPTF via multivalent interactions. Cell *145*, 692-706.

Shanle, E.K., Shinsky, S.A., Bridgers, J.B., Bae, N., Sagum, C., Krajewski, K., Rothbart, S.B., Bedford, M.T., and Strahl, B.D. (2017). Histone peptide microarray screen of chromo and Tudor domains defines new histone lysine methylation interactions. Epigenetics Chromatin *10*, 12.

Supplementary Figures

Supplementary Figure S1. Assay Optimization

Supplementary Figure S2. Background GST signal

Supplementary Figure S3. MPP8 chromo binding data

Supplementary Figure S4. CDYL2 chromo binding data

Supplementary Figure S5. CDYL2 western blot optimization and images

Supplemental Figure S6. L3MBTL3 3xMBT binding data

Supplemental Figure S7. 53BP1 TTD binding data

Supplemental Figure S8. Site-specific histone Kme antibody binding data

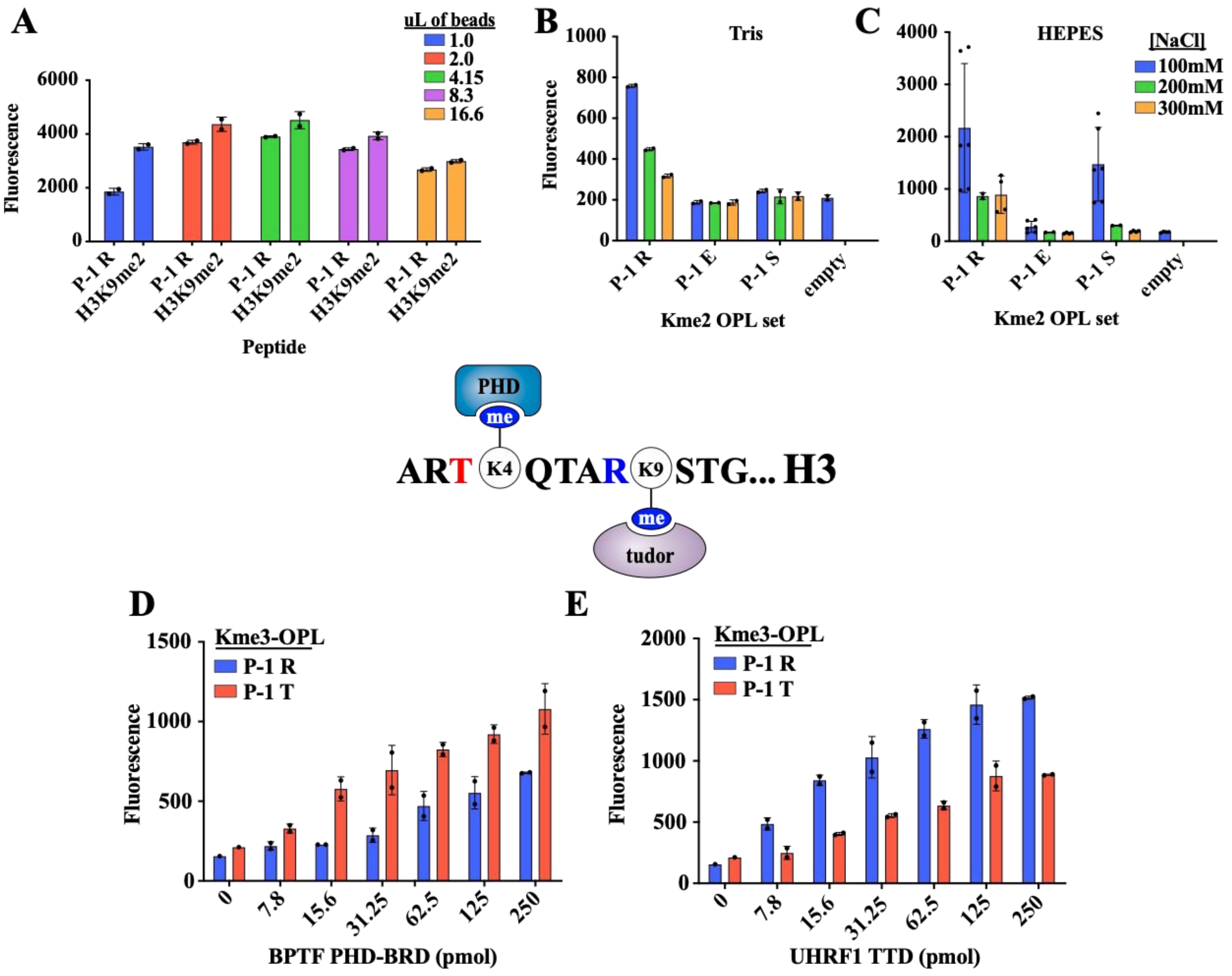

F

	1:5000	1:5000	1:10000	1:10000	1:20000	1:20000	1:40000	1:40000
1:2000	3003	3243	1770	1718	945	882	528	482
1:4000	3189	3199	1671	1758	877	904	508	443
1:8000	2695	2690	1511	1566	862	854	489	367
1:16000	2002	1985	1200	1261	694	707	424	358

1° GST sigma G7781		
2° Alexa488 rab invitrogen A11034		

Supplementary Figure S1. Assay Optimization. (**A**) Titration of bead amount with 150 pmol UHRF1 TTD-PHD with peptides P-1 R Kme3-OPL set or H3$_{1\text{-}15}$K9me2. Error is S.D. Titration of salt concentration with UHRF1 TTD and Kme2-OPL sets in (**B**) Tris and (**C**) HEPES buffers. Error is S.D. Titration of (**D**) BPTF PHD-Bromo and (**E**) UHRF1 TTD with the indicated Kme3-OPL sets. Error is S.D. (**F**) Simultaneous primary and secondary antibody titration using MPP8 chromo and Kme3-OPL set P-2 A. Dilutions for each antibody are on the axes and raw fluorescence is reported.

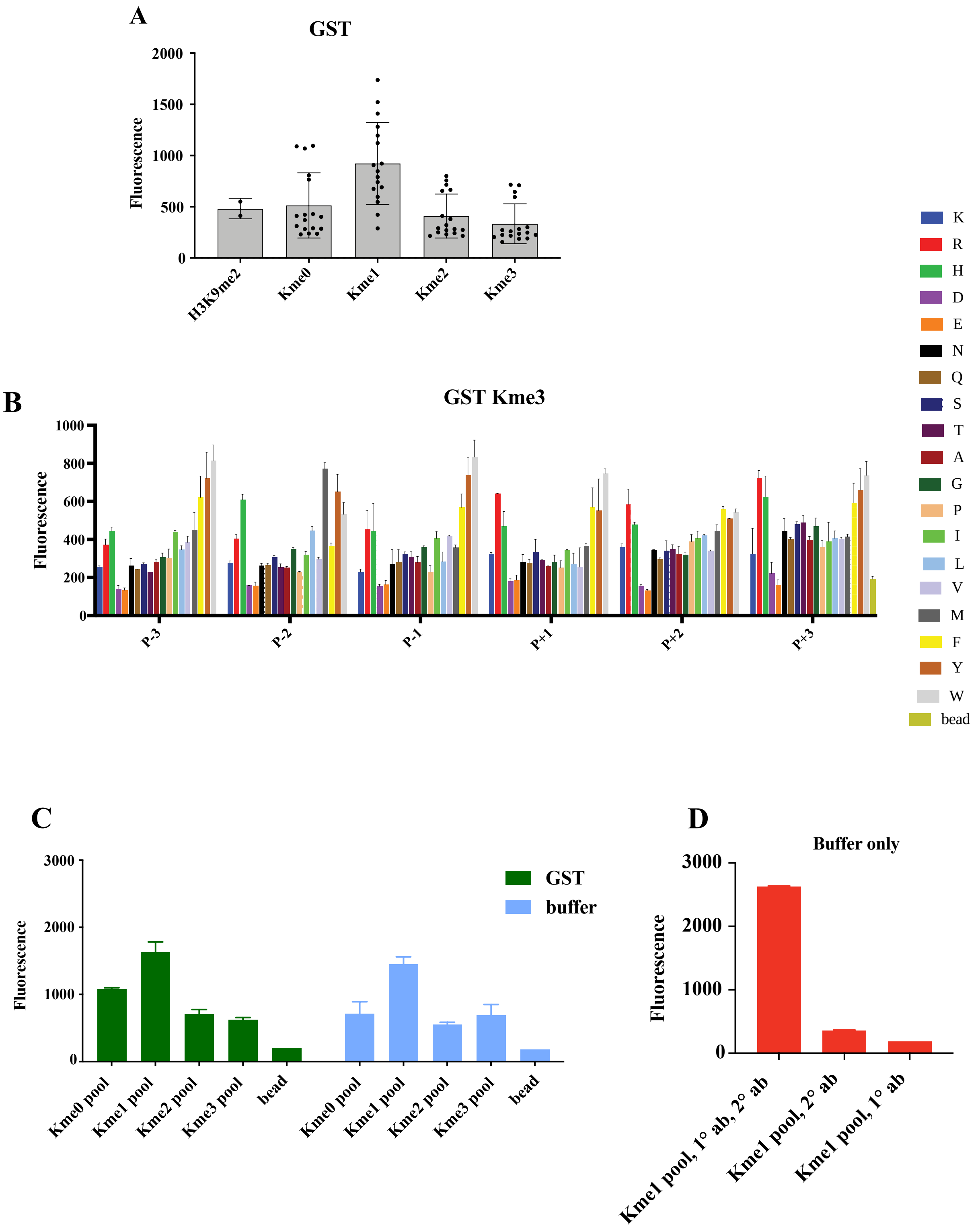

Supplementary Figure S2. Background GST signal. (**A**) All measurements of GST on Kme-OPL pools. Error is S.D. (**B**) GST interaction with Kme3-OPL sets. Data is plotted as an average of 2 replicate binding reactions. Error is S.D. (**C**) GST and Buffer alone on Kme-OPL pools. Data is plotted as an average of 2 replicate binding reactions. Error is S.D. (**D**) Buffer only with Kme1-OPL pool. Primary antibody dilution is 1:2000. Data is plotted as an average of 2 replicate binding reactions. Error is S.D.

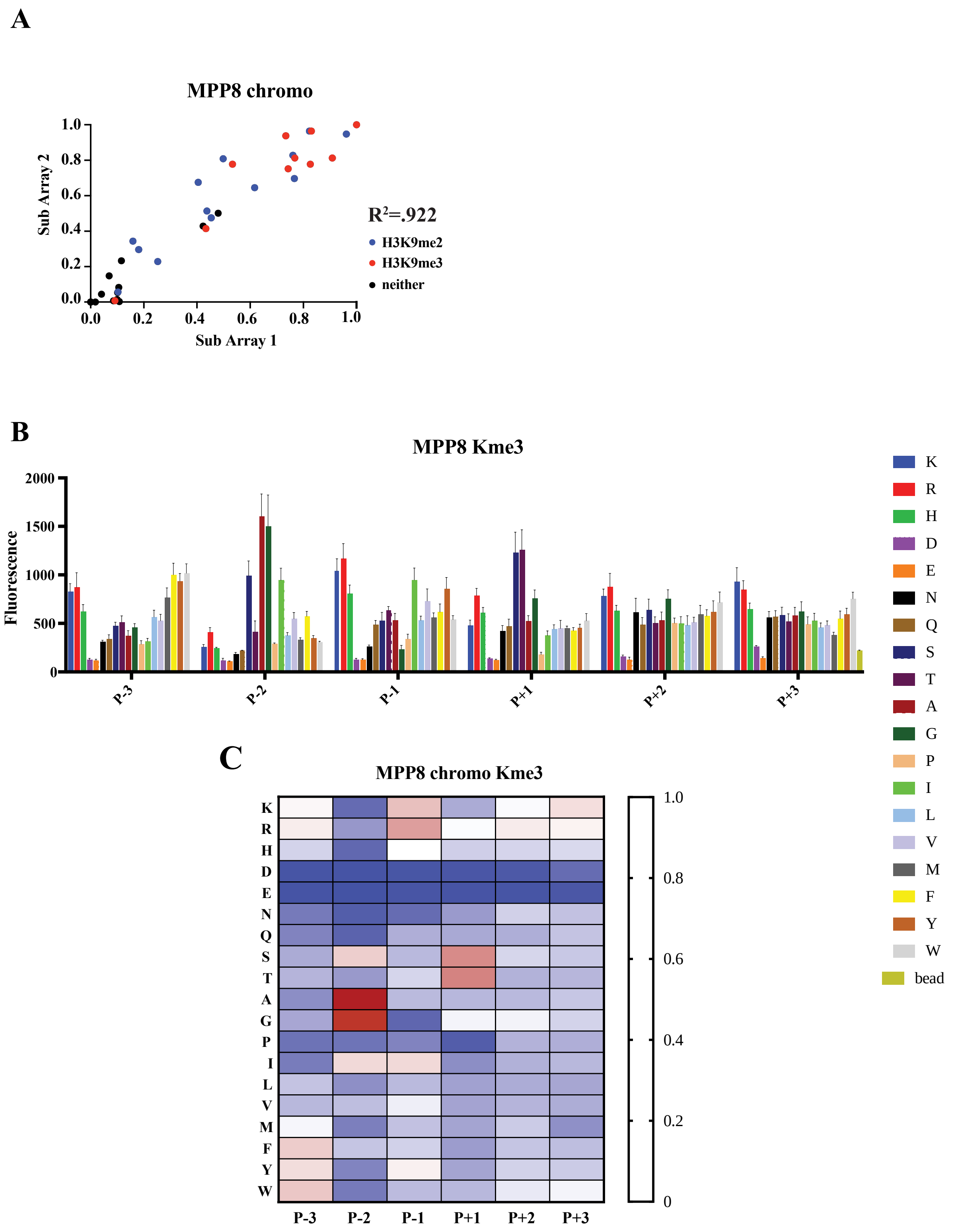

Supplementary Figure S3. MPP8 chromo binding data. (**A**) Scatter plot of normalized histone peptide array signals following hybridization with 1 μM MPP8 chromo. (**B**) MPP8 chromo interaction with Kme3-OPL sets. Data is plotted as an average of 4 replicate binding measurements. Error is S.D. (**C**) Heatmap is generated from normalized average Kme3-OPL set signals from 4 replicate binding measurements without GST subtraction.

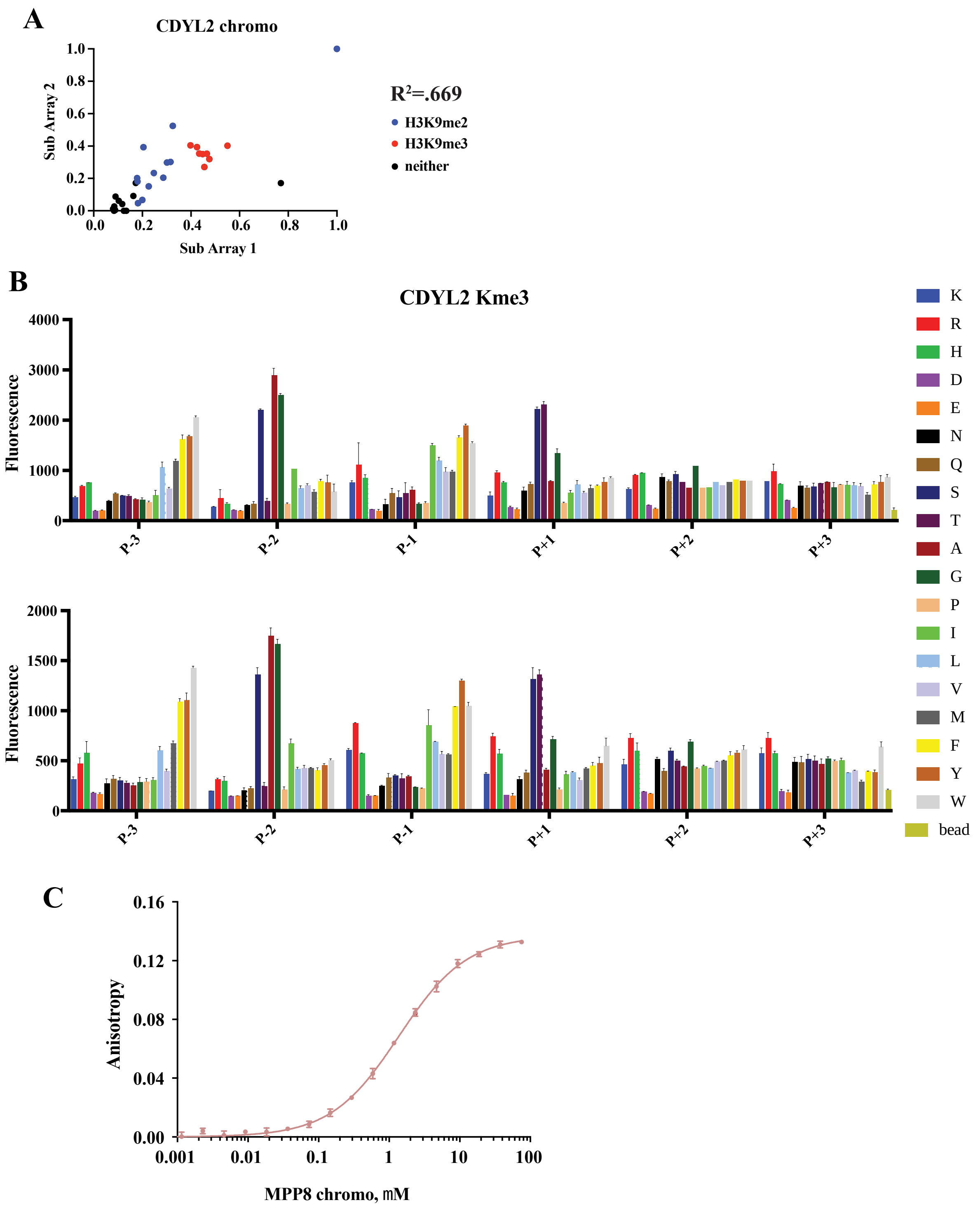

N-FAM peptides	purpose	K_d
GWAY(Kme3)TGKG	best CDYL2	K_d= 1.37 + 0.47μM

Supplementary Figure S4. CDYL2 chromo binding data. (**A**) Scatter plot of normalized histone peptide array signals following hybridization with 1 μM CDYL2 chromo. (**B**) CDYL2 chromo interaction with Kme3-OPL sets. Top and bottom data sets were collected independently and are both plotted as an average of 2 replicate binding measurements. Error is S.D. (**C**) MPP8 chromo fluorescence polarization. Data points are plotted as an average of 4 measurements from 2 independent experiments. Error is S.D.

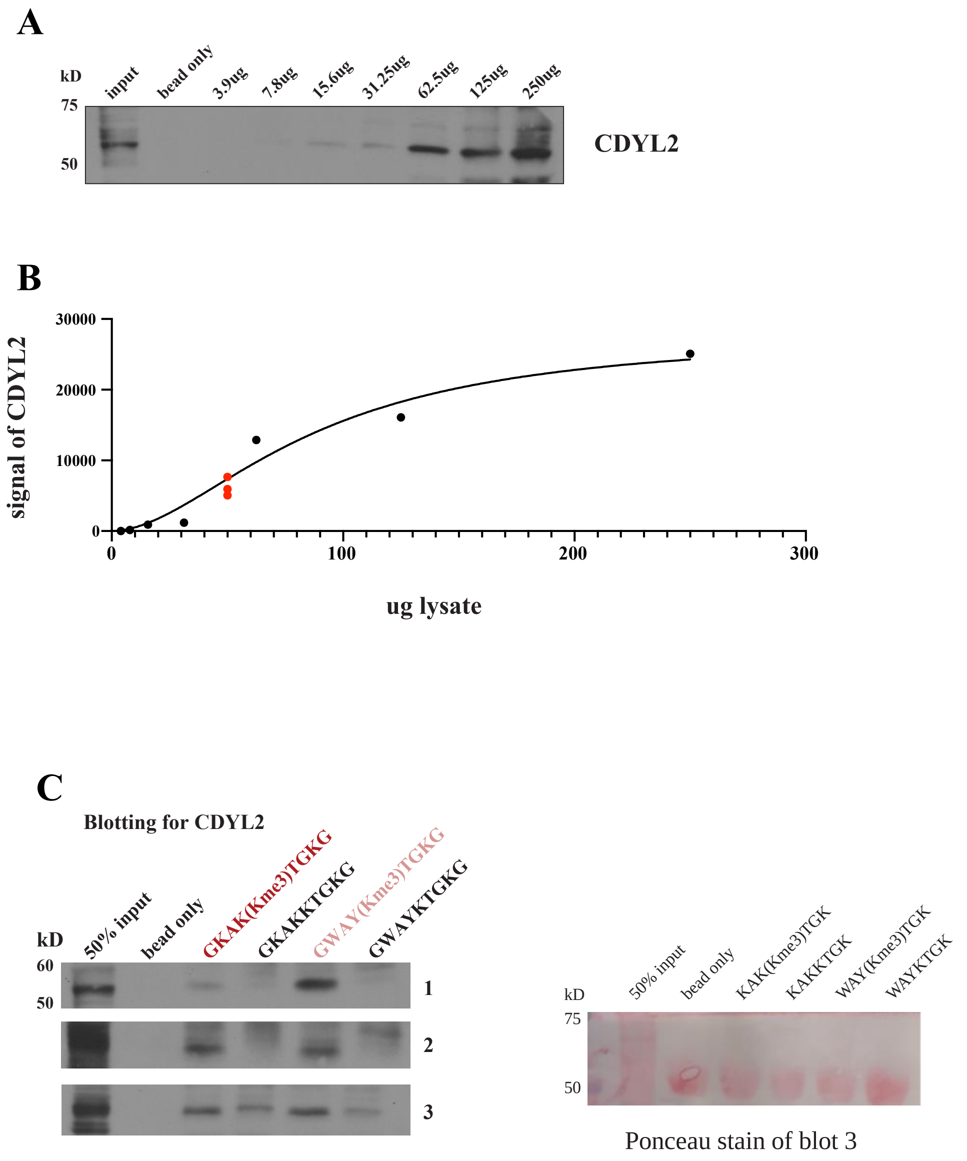

Supplementary Figure S5. CDYL2 western blot optimization and images. (**A**) Western blot of pulldowns with GWAY(Kme3)TGKG and the indicated amounts of HEK293 cell lysate. Input and bead only lanes were 25 µg lysate. CDYL2 antibody was used at a 1:1000 dilution. (**B**) Quantification of CDYL2 signal using Image J. Red dots are amounts of lysate and CDYL2 used in **C**. (C) Three blots from independent binding reactions using 50 µg of cell lysate. Ponceau staining is of blot 3.

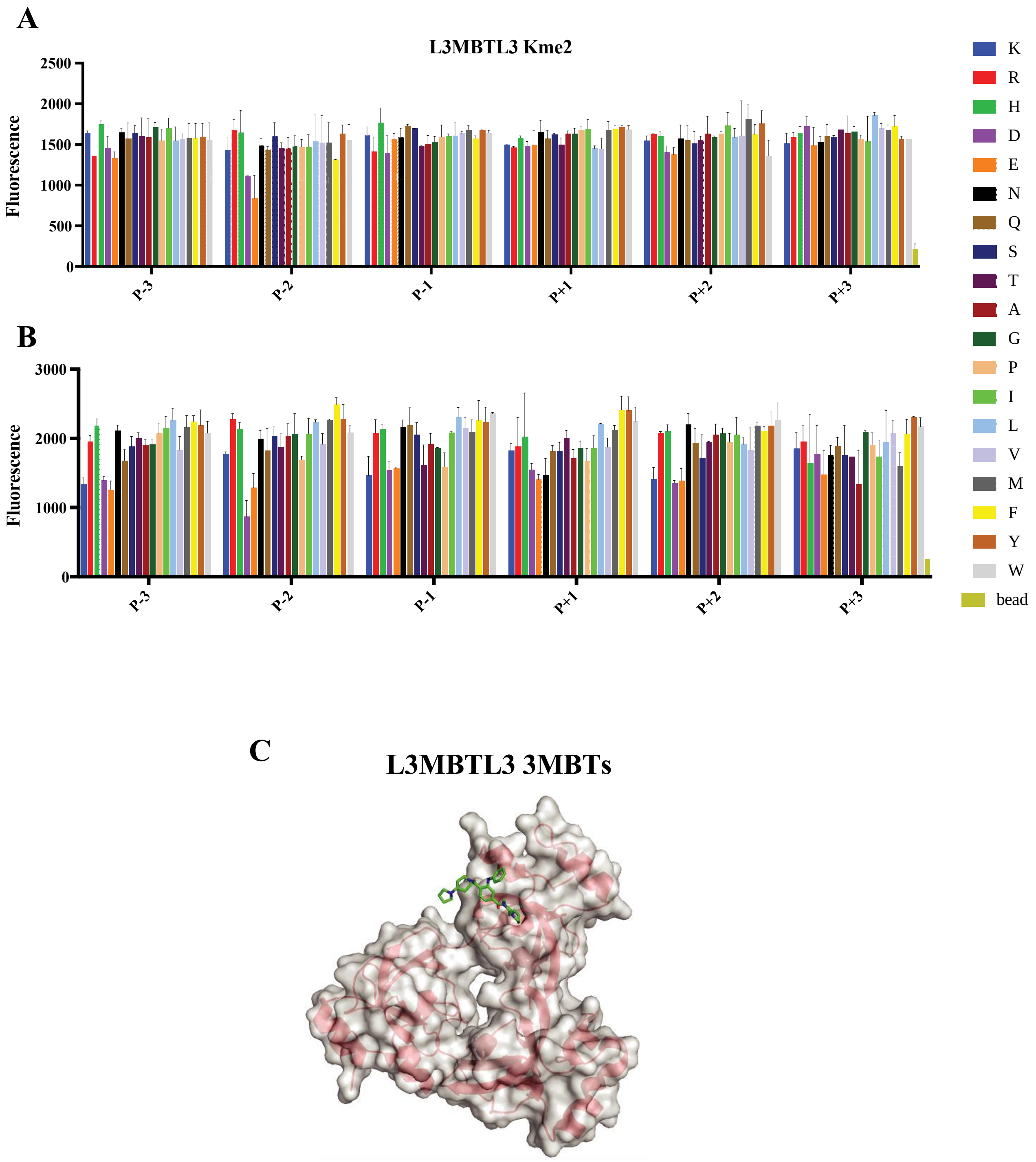

Supplemental Figure S6. L3MBTL3 3xMBT binding data. L3MBTL3 3xMBT interaction with Kme2-OPL sets from (**A**) duplicate or (**B**) triplicate binding measurements. Error is S.D. (**C**) L3MBTL3 3xMBT bound to UNC1215 (PDB:4FL6).

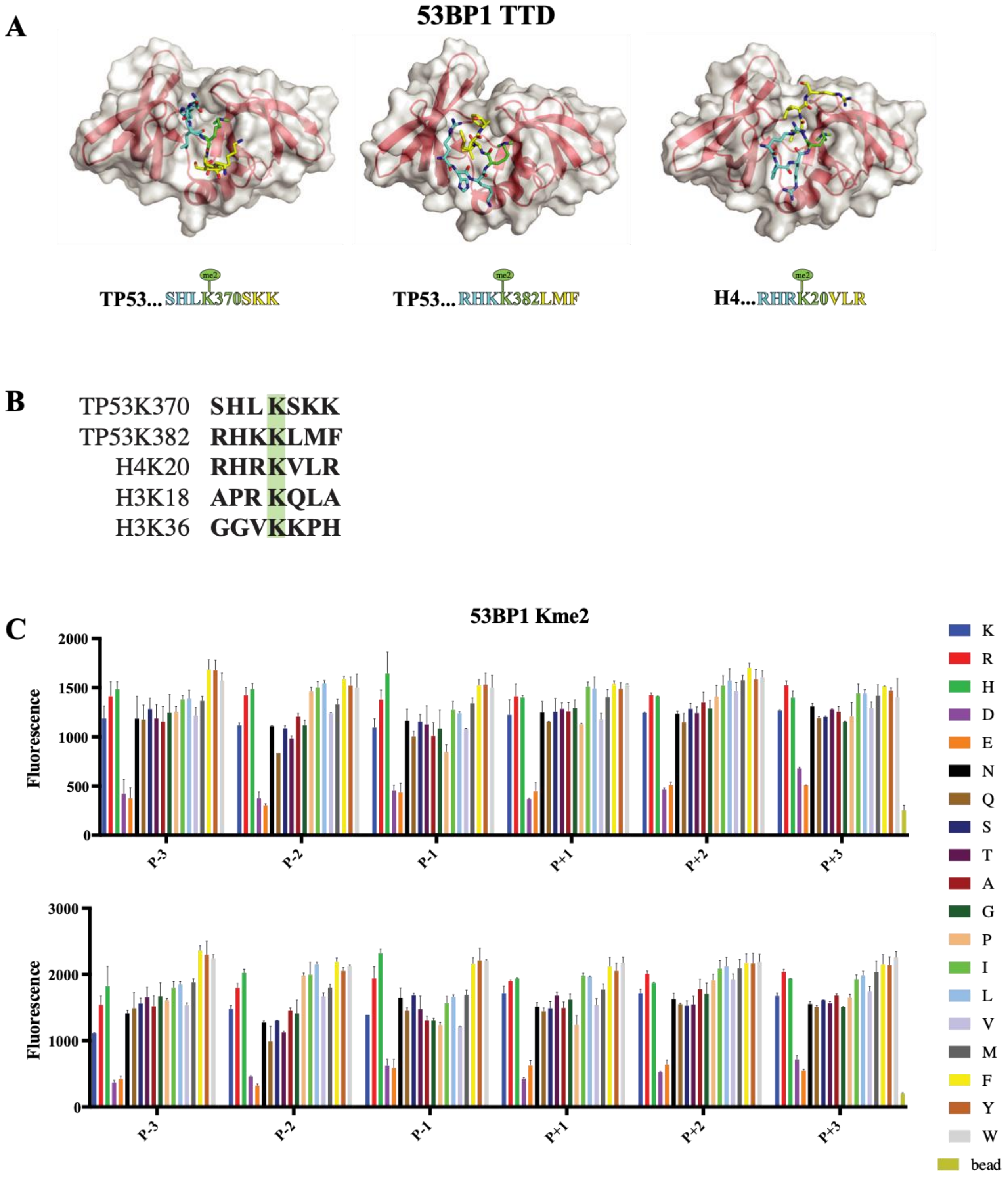

Supplemental Figure S7. 53BP1 TTD binding data. (**A**) Structures of 53BP1 TTD bound to p53K370me2 (PDB:2MWO), p53K382me2 (PDB:2MWP), and H4K20me2 (PDB:2LVM). (**B**) Sequences surrounding known Kme interactions of 53BP1 TTD. (**C**) 53BP1-TTD interaction with Kme2-OPL sets from duplicate binding measurements. Error is S.D.

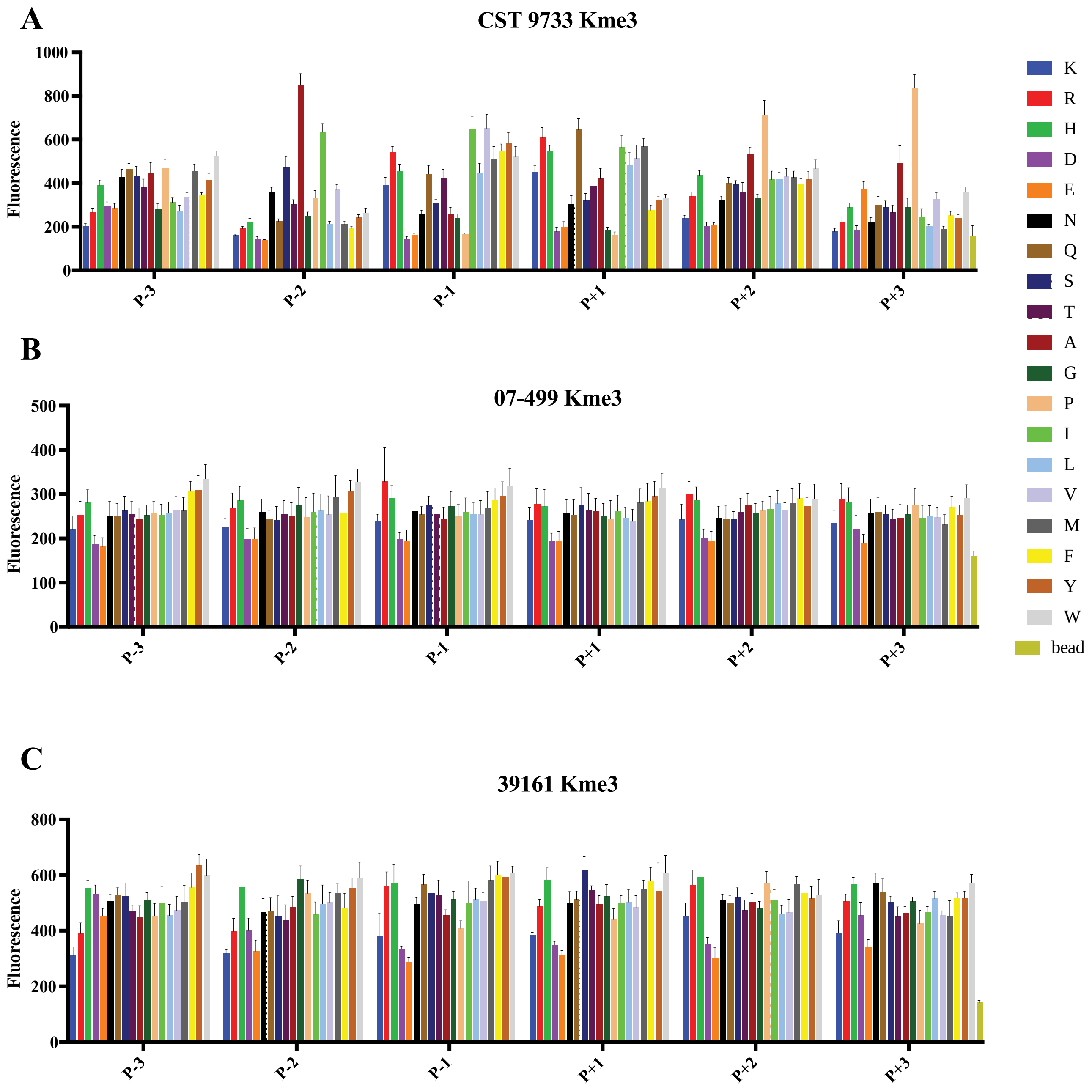
A
CST 9733 Kme3
Fluorescence
0
200
400
600
800
1000
P-3
P-2
P-1
P+1
P+2
P+3
B
07-499 Kme3
Fluorescence
0
100
200
300
400
500
C
39161 Kme3
Fluorescence
0
200
400
600
800
K
R
H
D
E
N
Q
S
T
A
G
P
I
L
V
M
F
Y
W
bead

D

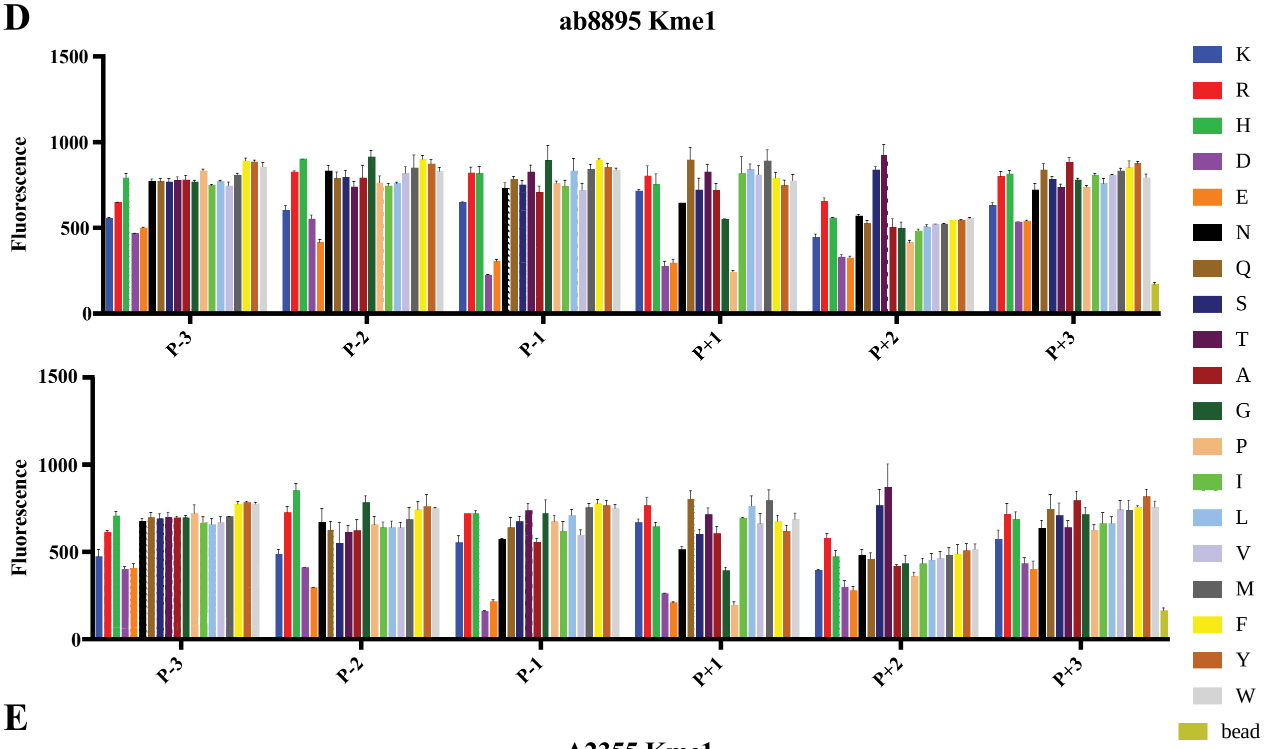

E

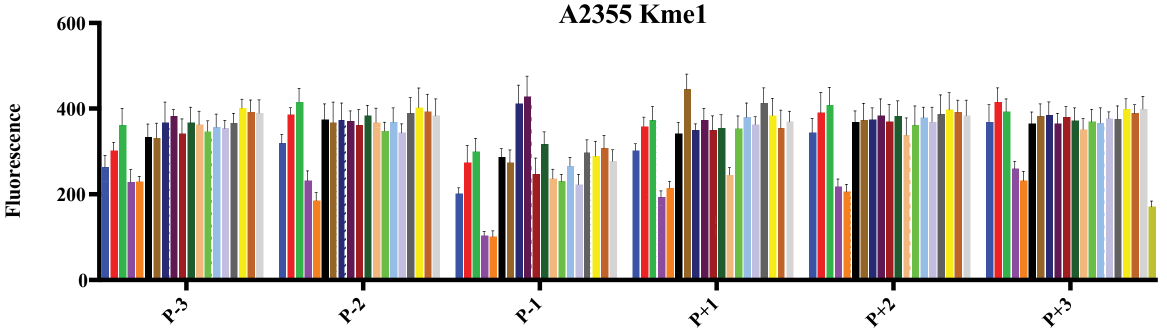

Supplemental Figure S8. Site-specific histone Kme antibody binding data. Interactions of the indicated antibodies with Kme-OPL sets. ab8895 data is from an average of duplicate binding measurements. All others are from 4 binding measurements. Error is S.D.
